# Prevalence of *Borrelia burgdorferi*-infected ticks from wildlife hosts, a response to Norris *et al*.

**DOI:** 10.1186/s13071-015-0739-z

**Published:** 2015-02-27

**Authors:** Maria D Esteve-Gassent, Abha Grover, Teresa P Feria-Arroyo, Ivan Castro-Arellano, Raul F Medina, Guadalupe Gordillo-Pérez, Adalberto A Pérez de León

**Affiliations:** Department of Veterinary Pathobiology, College of Veterinary Medicine and Biomedical Sciences, Texas A&M University, College Station, TX 77843 USA; Department of Biology, The University of Texas-Pan American, Edinburg, TX 78539 USA; Department of Biology, College of Science and Engineering, Texas State University, San Marcos, TX 78666 USA; Department of Entomology, College of Agriculture and Life Sciences, Texas A&M University, College Station, TX 77843 USA; Unidad de Investigación en Enfermedades Infecciosas, Centro Médico Nacional SXXI, IMSS, Distrito Federal, 06720 México; USDA-ARS Knipling-Bushland U.S. Livestock Insects Research Laboratory, Kerrville, TX 78028 USA

## Abstract

In a recent Letter to the Editor, Norris *et al.* questioned the validity of some of our data reported by Feria-Arroyo *et al*. The main issue investigated by us was the potential impact of climate change on the probable distribution of the tick vector *Ixodes scapularis* in the Texas-Mexico transboundary region. As an ancillary issue, an analysis of sequence data for the intergenic spacer of *Borrelia burgdorferi* was conducted. In the present letter, we provide further evidence supporting our original results, and advocate that extensive study of the population genetics of *B. burgdorferi* is needed in the Texas-Mexico transboundary region.

## Letter

We begin addressing the criticism expressed by Norris *et al*. [1] that our intergenic spacer (IGS) data for *Borrelia burgdorferi* sensu stricto sequences does not support the claim of *B. burgdorferi* tick infection rates in Texas and northeastern Mexico. First and foremost, perspectives like the one presented in our publication can help address the gap in knowledge of Lyme disease (LD) considering that its geographical area is expected to spread in the coming years [2]. Norris *et al*. [1] do not seem to dispute that we found *B. burgdorferi* in the *I. scapularis* collected in Texas and Mexico, but seem to question the tick infection rates reported. In their criticism Norris *et al.* [1] failed to relate our collective findings based on eco-epidemiological data documenting a continuum in the pathogenic landscape of LD in the Texas-Mexico transboundary region. In addition, members of our international research group collected nymph and adult *I. scapularis* from wild mammals in forest zones at an altitude of 1600–2670 meters above the sea level in the Mexican state of Nuevo León. This allowed us to state that northeastern Mexico meets ecological criteria to be considered endemic for LD [3].

The most relevant issue addressed by Feria-Arroyo *et al.* [4] consists of the potential impact of climate change in the distribution model of the tick vector *I. scapularis* in the Texas-Mexico transboundary region, based on present predictions of suitable habitat, as well as forecasting to year 2050. The major motivation of this study was the need to generate a current distribution model for this tick vector, together with future distribution models that forecast any changes under different climate change scenarios. These efforts will serve to increase our knowledge of the ecology of *B. burgdorferi* and its interaction with the competent vector *I. scapularis* in Southern US, where the incidence of LD in humans is very low. Norris *et al*. [1] disagree with the levels of *B. burgdorferi* infection that we detected on *I. scapularis* collected from non-human mammal hosts, although they do concur that *B. burgdorferi* infection does occur in Texas (http://www.cdc.gov/lyme/stats/). The evidence used by Norris *et al*. [1], to question our findings is based on data obtained by Williamson *et al.* [5]. Because the ticks on Williamson *et al*. study were obtained from humans in a passive surveillance setting, we do not consider a comparison of Williamson *et al*. [5] data and ours appropriate. Contrary to what Norris and collaborators wrongly state in their letter, our paper reported *B. burgdorferi* infection levels in ticks exclusively found in non-human-hosts or questing on vegetation. The Feria-Arroyo *et al.* report does not include any ticks recovered from humans. It is highly possible that infection levels on non-human hosts differ from infection levels detected in humans, thus making both datasets not comparable.

Also, Norris *et al*. [1] affirm that “tick stage was not reported” for our collections, but this information is addressed extensively in page 12, paragraph 2, where collected adult ticks are described. Furthermore, they assert that infection prevalence in these ticks removed from animal hosts would likely be different from that of questing nymphs that are more likely to infect humans, further supporting our argument for the incompatibility of contrasting these two datasets, ours and the one in Williamson *et al.* [5]. It is worth mentioning that Norris *et al.* [1] close that paragraph stating that our study “as designed, would not provide a clear-cut indication of the human risk for Lyme disease”, thus implying a goal that was never mentioned in the Feria-Arroyo *et al.* [4] paper. Nowhere in our paper do we address human risk for LD in the Texas-Mexico transboundary region, nor do we make any implications or conclusions in that direction. Our main model results are restricted to a predicted distribution for *I. scapularis,* which is based on a presence-absence model with no assumptions being made on the density of the different tick life stages. Moreover, we clearly indicate the need for further comparative studies to better understand LD in different ecological regions.

**Figure 1:**
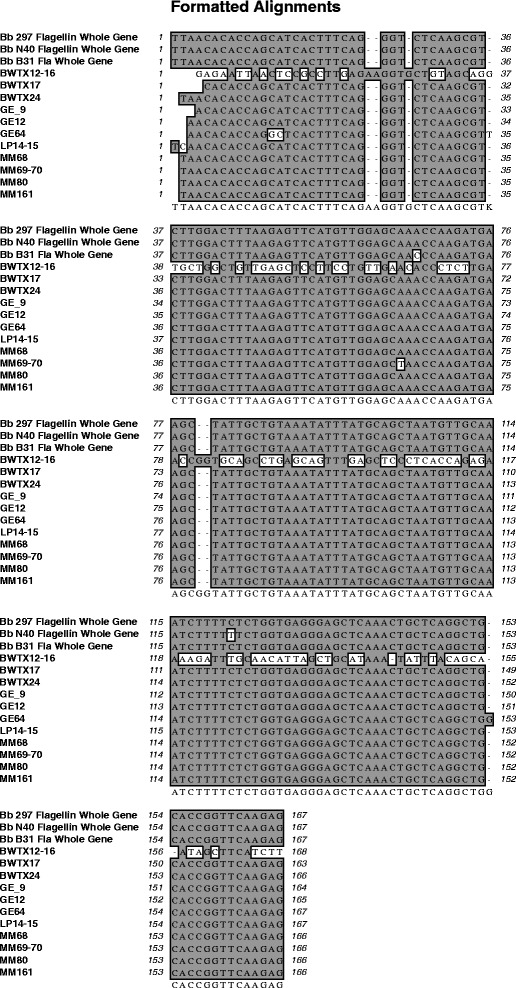
**Multiple alignments for the** ***flaB*** **DNA sequence analysed from samples collected in the study presented by Feria-Arroyo** ***et al.*** **[** 4 **]** **together with 5 more samples acquired from the same regions in Texas in different times of the year.** In the alignment the corresponding *flaB* sequences from *B. burgdorferi* strains B31, N40 and 297 were used as reference. Sequences were aligned using MacVector 13.0.7 (MacVector Inc., North Carolina). GE, LP and MM samples correspond with GEWMA, LPWMA and MMWMA samples.

Infection levels using a second genetic marker (*flaB),* confirmed the results originally obtained by the16SrRNA- 23SrRNA gene intergenic spacer (IGS) of *B. burgdorferi*. To test for *B. burgdorferi* infection using *flaB*, a total of 11 tick DNA samples were analyzed by PCR following the same protocol described in Feria-Arroyo *et al.* [4]. For this new analysis, we could only utilize samples from which we had remaining aliquots to perform the *flaB* PCR and sequencing reactions (samples BWTX12-16, BWTX17, BWTX24, GEWMA9, GEWMA12 and GEWMA64). We also added samples collected from white tailed deer (WTD) and gemsbok (samples LPWMA14-15, MMWMA68, MMWMA69-70, MMWMA80 and MMWMA161) from the same locations used in our original study, but harvested at a later time point. Thus, all tick samples used were collected from wildlife. Two hundred and thirty nucleotide amplicons were submitted for sequencing using forward and reverse primers for *flaB* [6]*.* Sequences were cleaned individually before assembly using MacVector Version 13.0.7 (MacVector Inc., North Carolina) as follows. First, the 5’- and 3’- ends were removed from each sequence to avoid utilization of unclean and noisy sections obtained during sequencing. After cleaning the ends, each peak in the chromatograms was checked for accuracy of the corresponding nucleotide to make sure listed nucleotides were correct. Once all sequencing results were cleaned, the forward and reverse sequences were assembled using MacVector Assembler 13.0.7 (MacVector Inc., North Carolina). The consensus sequence produced was then used for further alignment analyses with the same *B. burgdorferi* control strains utilized by Norris *et al*. [1] (B31, N40 and 297) using MacVector 13.0.7 (MacVector Inc., North Carolina). As shown in Figure 1, the Texan *flaB* sequences show very low variability. This low variability was also observed when IGS sequences were used. Samples GEWMA64, MMWMA69-70, MMWMA68 and MMWMA161 show the highest variability (16, 2, 10 and 10 nucleotide changes respectively). On the other hand, BWTX12-16, a questing tick, has a significantly different sequence from either of the controls or the other Texan samples, suggesting that the degree of genetic variation of *B. burgdorferi* in the regions sampled likely exceed the values found by Feria-Arroyo *et al.,* study [4], which only sampled a limited number of potential vertebrate hosts.

**Figure 2:**
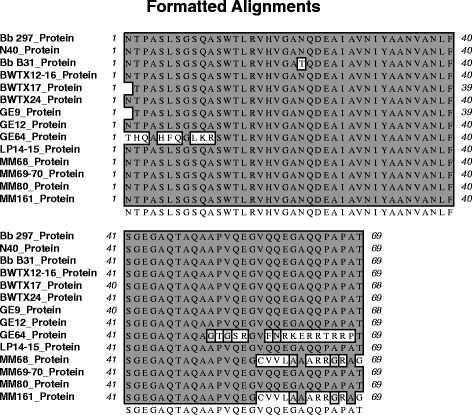
**Multiple alignments for the FlaB amino acid sequence corresponding to the fragment amplified in this study.** The corresponding FlaB amino acid sequences from *B. burgdorferi* strains B31, N40 and 297 were used as reference. Sequences were aligned using MacVector 13.0.7 (MacVector Inc. North Carolina). All sequences were identified as *B. burgdorferi* FlaB sequences when analyzed using BLAT® (MacVector 13.0.7, MacVector Inc., North Carolina). GE, LP and MM samples correspond with GEWMA, LPWMA and MMWMA samples.

Norris *et al*. [1] argued that the infected ticks reported in our study were found infected with *B. burgdorferi* likely due to contamination of the PCR reactions with DNA from the strain B31 of *B. burgdorferi*, the positive control used in the study. Nevertheless, *B. burgdorferi* B31 *flaB* gene has a cytosine (C) at position 75 in this alignment (Figure 1) while the Texas isolates had an adenine (A). The A in the Texas isolate makes them more similar to strains N40 and 297 than to B31. Contamination of our samples with strains N40 and 297 is impossible, since these strains are not present in the laboratory in which molecular analyses were carried out. The codon in which this nucleotide change was detected translates to Asparagine (Asn) in strains N40, 297 and in the Texan strains, and to Threonine (Thr) in the B31 strain (Figure 2). To date, no population genetic studies of *B. burgdorferi* have been conducted in the Texas-Mexico transboundary region, or in southern US. Consequently, the level of genetic variation within *B. burgdorferi* and its tick vector is unknown. In a recent study, Clark *et al*. [7] detected *B burgdorferi* sensu stricto in four human patients from Texas. In this study, the authors compared the *flaB* sequence amplified from human Lyme patients across the country and showed that *flaB* sequences in Texas are very similar to that of the B31 strain. Interestingly, the *flaB* sequences found in our study in non-human hosts differed from the B31 strain, yet both we [4] and Clark *et al*. [7] found low sequence variability in the *flaB* gene within samples collected in Texas.

**Figure 3:**
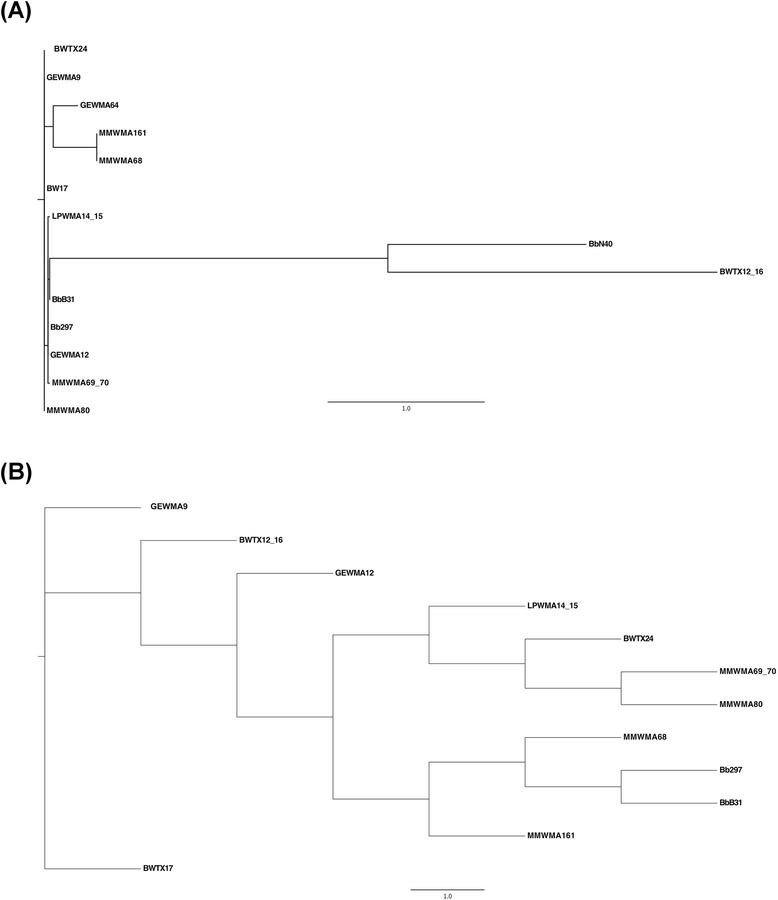
**Neighbor-Joining phylogenetic tree of the** ***flaB*** **sequences analysed in this study.** For the phylogenetic analyses, ClustalW2 [8] generated preliminary multiple sequence alignments for both the DNA sequences **(A)** and their respective putative amino acid sequences (**B**, obtained through the NCBI’s blastx program). These alignments were fed into jModeltTest 2.1.7 [9] and ProtTest 3 [10], programs that perform maximum likelihood (ML) optimization to assess the best-fit models for nucleotide substitution and amino acid substitution, respectively. These results informed the parameter sets to be used in RAxML v1.1 [11], a randomized, accelerated ML phylogenetic tree search program. These ML searches were conducted through 100 inferences of 100 distinct, randomized trees using the general time-reversible (GTR) model with gamma distributed rate heterogeneity for nucleotide data and the LG model with rate heterogeneity for amino acid data. The phylogenetic trees were visualized using FigTree v1.4.2 [12]. Figure Xa demonstrates that BWTX12-16 possesses a widely divergent nucleotide sequence from that of the other sequences, yet Figure Xb shows that the BWTX12-16 amino acid sequence is nested with the other sequences.

In order to further verify that the nucleotide differences we observed between N40, 297, the Texas samples and B31 were not due to sequencing error, we evaluated whether or not they encode for a functional FlaB polypeptide. To this end, the translated sequences were generated. As shown in Figure 2, none of the samples analysed contained stop codons. Moreover, those containing higher sequence variability (MMWMA69-70, MMWMA68, MMWMA161, and BWTX12-16), translated into functional FlaB polypeptides with very limited amino acid changes. Furthermore, a phylogenetic tree (Figure 3) was generated and the Texas samples grouped with the 297 strain rather than with the B31 strain, suggesting that our previous results were unlikely the result of strain contamination of PCR reactions and unlikely caused by poor sequencing quality.

Taken together all the analyses generated throughout our studies, and previous studies with human isolates of *B. burgdorferi* sensu stricto from Texas [7], suggest little genetic variability in the markers analyzed (*flaB* and IGS) and support the fact that contamination during the testing process was unlikely. The *flaB* sequences reported in this letter have been published in GenBank (NCBI National Center of Biotechnology Information, accession numbers KM875668 through KM875675, http://www.ncbi.nlm.nih.gov/genbank/).

Norris *et al*. stated in their letter that due to the low variability observed in the IGS from the Texas samples most, if not all of them, were likely to have been originated from the same clone which they assume could be the product of contamination with the B31 strain. We disagree with the interpretation put forward by Norris *et al*. and instead think it is more likely that the lack of variability reported in Feria-Arroyo *et al*. reflects the level of *B. burgdorferi* variability present in the Texas-Mexico transboundary region. Several of the ticks included in the Feria-Arroyo study were collected from white-tailed deer, gemsbok and dog. These mammalian hosts, particularly white-tailed deer, harbour ticks from several lineages. Thus, ticks collected from white-tailed deer, even if collected from the same individual, are likely to carry a representation of the *B. burgdorferi* strains present in a particular location. Thus, the *B. burgdorferi* genetic diversity reported by Feria-Arroyo *et al*., likely represents the genetic variation present in the Texas-Mexico transboundary region. Interestingly, our host-seeking (i.e., questing) ticks found on vegetation (i.e., BWTX12-14, 17 and 24) show more genetic variation than samples collected from white-tailed deer and gemsbok, yet samples from questing ticks in the Texas-Mexico transboundary region show still lower variability than what has been reported in the US Northeast and Midwest [13].

The lack of variation in the *IGS* and *flaB B. burgdorferi* sequences may reflect the fact that low genetic variation of *B. burgdorferi* is present on ticks feeding on non-human hosts in the Texas-Mexico transboundary region sampled areas. It is not uncommon for bacteria to show different levels of genetic variation at different geographic locations [14,15]. Variation in sequence diversity among different geographic regions can be explained by different selection regimes, local adaptation, recent colonization events, and/or genetic drift. Currently several ecological factors associated with LD transmission in the Texas-Mexico transboundary region are unknown. Without further research on the ecology of competent and potential vectors and hosts, it is difficult to pinpoint the reasons for the variability observed in *IGS* and *flaB* sequences with a high degree of accuracy. The fact that *B. burgdorferi* sequences differ widely in genetic diversity between *B. burgdorferi* samples collected from ticks feeding on non-human and human-hosts from different regions is a clear indication that this needs further research. Although this was not the main topic of our paper we conclude that additional studies should explore the reasons explaining the observed *IGS* and *flaB B. burgdorferi* sequence homogeneity in the Texas-Mexico transboundary region [4,7] as compared with what has been observed in the Northeastern and Midwestern US.

Our team supports the fact that Texas is a low incidence state for human cases of LD [16-19]; however, the ecology of this disease in the Texas-Mexico transboundary region is understudied. Given that this region is a completely different environment, as compared to Northeastern and Midwestern US, it is highly likely that LD ecology will follow a different dynamic. Variation in tick ecology, tick questing behaviour, and vertebrate host communities between these regions that have considerable differences in climate, species diversity and composition, are likely to influence the ecology of LD as well as its transmission potential to humans. Thus, extrapolations based on findings from the Northeastern and Midwestern US may not be accurate enough to understand LD transmission and ecology in other regions. Our research team is interested in elucidating the ecological factors that explain the low human infection incidence reported in the southern US and northern Mexico to better understand the dynamics of this tick borne disease across its distribution range.

Norris *et al*. suggest that the Feria-Arroyo *et al.* [4] publication is advocating a high LD risk in Texas and Mexico but this cannot be further from the truth. Unlike publications that have aimed to create disease risk maps based on questing infected nymphs [16,17,19] the Feria-Arroyo *et al.* [4] presents a habitat suitability model for the presence of the vector in the studied area, and no predictions are made in regards to the density and prevalence of infected ticks. We agree with Norris *et al*. that it is unfortunate that the “Feria-Arroyo *et al*. [4] has been publicised as an indication of a significant Lyme disease risk in Texas” but misinterpretations of that paper in media outlets are not the responsibility of the authors. Throughout the Discussion section of Feria-Arroyo *et al.* [4] it is stressed that a direct correlation between vector presence and human disease cannot be made because of the lack of knowledge about the disease ecology for this pathogen in the studied region.

Norris *et al.* go a step further and even ask for the paper to be removed from the literature, but we claim this move is unwarranted for two main reasons. First, it will be naïve to think that a whole public health strategy will be swayed based on a study that does not address human risk for the disease. Secondly, the main findings of the Feria-Arroyo *et al*. paper [4], which are the vector distribution models, remain unchanged independently of the disputed *B. burgdorferi* prevalence rates. Removing the contribution of these vector distribution models from the literature would be a step backwards for the continued study of tick-borne illnesses in the southern United States, a goal that we agree with Norris *et al*. is worthy of further academic work.
